# miR-146-5p restrains calcification of vascular smooth muscle cells by suppressing TRAF6

**DOI:** 10.1515/med-2022-0471

**Published:** 2022-09-24

**Authors:** Jing Yang, Xiaoman Zhou, Jingwei Lu, Meng Li

**Affiliations:** Department of Cardiology, The Fourth Hospital of Harbin Medical University, Harbin 150001, Heilongjiang, China; Department of Radiology, Wuhan Pulmonary Hospital, Wuhan 430030, Hubei, China; Department of Physical Examination, The Fourth Hospital of Harbin Medical University, Harbin 150001, Heilongjiang, China; Department of Cardiology, The Fourth Hospital of Harbin Medical University, 37 Yiyuan Street, Nangang District, Harbin 150001, Heilongjiang, China

**Keywords:** miR-146-5p, TRAF6, vascular calcification, atherosclerosis

## Abstract

Vascular calcification is a prominent manifestation of advanced atherosclerosis. Tumor necrosis factor-receptor-associated factors (TRAFs) were reported to participate in atherosclerosis development. In this study, the role and mechanism of TRAF6 in vascular calcification were explored. To induce the vascular calcification, oxidized low-density lipoprotein (Ox-LDL) was applied to treat vascular smooth muscle cells (VSMCs). TRAF6 protein expression in VSMCs was assessed by western blotting. Osteogenic differentiation of VSMCs was assessed by alkaline phosphatase activity analysis. Mineral deposition in VSMCs was evaluated by von Kossa staining. VSMC proliferation, migration, apoptosis, inflammation, and reactive oxygen species (ROS) generation were detected using cell counting kit-8, Transwell, flow cytometry, reverse transcriptase quantitative polymerase chain reaction (RT-qPCR), and dichlorodihydrofluorescein diacetate staining, respectively. Luciferase reporter assay was utilized to identify the binding relationship between miR-146-5p and TRAF6 in VSMCs. We found that Ox-LDL administration induced the calcification of VSMCs and elevated the TRAF6 level. TRAF6 knockdown restrained VSMC calcification, proliferation, migration, inflammation, and ROS generation caused by Ox-LDL. Mechanically, TRAF6 was targeted by miR-146-5p in VSMCs. Furthermore, TRAF6 overexpression offset the inhibitory effects of miR-146-5p upregulation on vascular calcification in VSMCs under the Ox-LDL condition. Overall, miR-146-5p restrains the calcification of VSMCs by suppressing TRAF6.

## 1 Introduction

Atherosclerosis is a chronic inflammatory disease and a major cause of coronary heart disease, cerebral infarction, and peripheral vascular disease [1]. Atherosclerosis is characterized by the deposition of blood components (e.g., lipids and complex sugars), the elevation of collagen fibers, the proliferation of vascular smooth muscle cells (VSMCs), and the intima of arteries [2]. The etiology and pathogenesis of atherosclerosis are complex and multiple risk factors contribute to atherosclerosis, such as hyperlipidemia, hypertension, excessive smoking, diabetes, obesity, immune impairment, and genetic factor [3,4]. At present, beta-blockers, lipid-lowering therapy, gene therapy, and surgery are the main choices for atherosclerosis treatment [5]. However, atherosclerosis causes a series of cardiovascular diseases including acute ischemic stroke, which has imposed a heavy nursing care burden on people [6,7]. Thus, finding more effective therapies for patients with atherosclerosis are very urgent.

VSMCs are the main cells in the media of blood vessels. The abnormal proliferation and migration of VSMCs are important biological events during the development of atherosclerosis [8]. The inflammatory response is an important trigger for the phenotype switch of VSMCs [9]. Vascular calcification is an active process resembling bone formation and involves osteogenic differentiation of VSMCs in response to oxidative stress, inflammatory cytokine release, calcium deposition, and altered extracellular matrix [10,11,12,13]. Oxidative stress has been identified as one of the potent factors involved in the regulation of vascular calcification [14]. Low-density lipoprotein (LDL) is a lipoprotein particle that carries cholesterol into peripheral histiocytes and can be oxidized into oxidized LDL (Ox-LDL) [15]. Ox-LDL is a crucial risk factor for atherosclerosis progression and affects the proliferation and migration of VSMCs [16]. Ox-LDL plays a key role in inducing the osteogenic differentiation and calcification of VSMCs. It is important to elucidate the mechanisms by which Ox-LDL modulates VSMC calcification and other phenotypes.

Tumor necrosis factor (TNF) receptor-associated factor 6 (TRAF6) is a member of TRAF protein family [17]. TRAF6 acts downstream of cytokine and toll-like receptors (TLRs) to regulate the activation of nuclear factor kappa-light chain enhancer of activated B cells (NF-κB) [18], which plays a key role in the progression of inflammation-related diseases [19]. Accumulating studies have demonstrated that TRAF6 participates in atherosclerosis progression. Small-molecule inhibitors that block the interaction between CD40 and TRAF6 (TRAF-STOPs) overcome the current limitations of long-term CD40 inhibition in atherosclerosis, which has the potential to become a future therapeutic for atherosclerosis [20]. Endothelial TRAF6 deficiency reduced atherosclerosis in female ApoE(−/−) mice by inhibiting NF-κB-dependent proinflammatory gene expression and monocyte adhesion to endothelial cells [21]. Furthermore, TRAF6 plays an important role in calcification during the progression of intervertebral disc degeneration [22].

In this study, we hypothesized that TRAF6 regulates VSMC calcification and other phenotypes. We also investigated the mechanisms related to the role of TRAF6, which might offer novel ideas for treating patients with atherosclerosis.

## 2 Material and methods

### 2.1 Bioinformatic analysis

Potential upstream miRNAs (miR-194-5p, miR-146-5p, miR-124-3p, miR-125-5p, miR-506-3p, and miR-140-3p) of TRAF6 and a binding site of miR-146-5p in TRAF6 3′UTR were predicted by Targetscan (http://www.targetscan.org/).

### 2.2 Cell culture

Human VSMCs were purchased from Thermo Fisher Scientific (Waltham, Massachusetts, USA) and cultured in Dulbecco’s modified Eagle’s medium (Gibco, NY, USA) containing 10% foetal bovine serum, streptomycin (100 μg/mL), and penicillin (100 U/mL) (Gibco) at 37°C with 5% CO_2_. The cultured VSMCs at passages 3–6 were used for subsequent experiments.

### 2.3 Isolation and oxidation of Ox-LDL

As previously described [23], 1.019–1.063 g/mL of LDL was isolated from human plasma (F0150-100 mL; Jianglai Biotechnology, Shanghai, China) by sequential density gradient ultracentrifugation. After dialysis, 5 µM CuSO_4_ was incubated with separated LDL fraction to oxidize LDL. Next, 20 µM EDTA was used to stop the oxidation. Finally, LDL oxidative degree was measured by thiobarbituric acid-reactive substance using 365-nm wavelength and Ox-LDL containing 16.8 ± 0.58 nmol/mg of thiobarbituric acid-reactive substance was used in this study.

### 2.4 Cell treatment and transfection

After cell culture, human VSMCs (2 × 10^5^ cells/well) were seeded in a six-well plate. The cells were treated with Ox-LDL (50 mg/mL) or native LDL (N-LDL) (50 mg/mL) in the presence of 10 mM beta-glycerophosphate for 7 days. For cell transfection, TRAF6 small-interfering RNA (si-TRAF6) was used to knockdown TRAF6 with si-NC as a negative control. miR-146-5p mimics were used to upregulate miR-146-5p with NC mimics as negative controls. Coding regions of TRAF6 were subcloned into the pcDNA3.1 vector to elevate TRAF6 expression with empty pcDNA3.1 as a negative control. All plasmids or oligonucleotides mentioned above were purchased from Genechem (Shanghai, China). Afterward, cell transfection was conducted using Lipofectamine 2000 (Invitrogen, USA) and the transfection efficiency was examined using RT-qPCR after 48 h.

### 2.5 Reverse transcriptase quantitative polymerase chain reaction (RT-qPCR)

TRIzol reagent (Invitrogen) was utilized to isolate total RNA from human VSMCs. AMV Reverse Transcriptase (Roche, Germany) was applied to reverse transcribe the total RNA into cDNA. RT-qPCR was performed using SYBR Green PCR Master Mix (Applied Biosystems, USA) on an ABI 7500 Real‐Time PCR system (Applied Biosystems). The internal reference of TRAF6 and miRNAs were GAPDH and U6, respectively. The results were analyzed using the 2^−ΔΔCt^ method.

### 2.6 Western blotting

The concentration of total protein from human VSMCs was measured by BCA protein assay kit (Pierce, USA). Protein samples were separated by 10% sodium dodecyl sulfate-polyacrylamide gel electrophoresis; and transferred to polyvinylidene difluoride (PVDF) membrane. PVDF membrane was blocked with 5% nonfat dry milk and incubated with primary antibodies including anti-TRAF6 (ab33915; 1:500; Abcam) and anti-GAPDH (ab8245; 1:1,000; Abcam) at 4°C overnight, followed by incubation with secondary antibody horseradish peroxidase-labeled IgG (Life Technologies) at room temperature for 2 h. The protein bands were visualized with enhanced chemiluminescence reagent (Bio-Rad) and analyzed with ImageJ software.

### 2.7 Calcification assay

To induce calcification, 10 mM beta-glycerophosphate was used to treat human VSMCs under Ox-LDL condition for 7 days. According to the manufacturer’s instructions, von Kossa staining kit (GENMED, Shanghai, China) was used to detect mineral deposition in human VSMCs after cell treatment and transfection. As previously described [24], *O*-cresolphthalein complexone method was used to assess calcium content. After washing with phosphate buffer saline (PBS), human VSMCs were treated with 0.6 N HCl for decalcification for 24 h. Then, protein content served as a normalization to calcium content. Alkaline phosphatase (ALP) [25] activity assay was carried out as previously described [15]. Human VSMCs with different treatments were collected with 0.1% Triton X-100 in PBS at 3 and 7 days. BCA protein assay kit was applied to quantify the concentration of protein in human VSMCs. After protein samples were incubated with 180 µL p-NPP substrate at 37°C for 15 min, NaOH (50 µL; 3 M) was added to the mixture. Absorbance was then measured at 405 nm and ALP activity was presented as nmol/mL *p*-nitrophenol converted per microgram of protein per minute.

### 2.8 Cell counting kit‐8 (CCK‐8) assay

VSMCs were seeded in a 96-well plate (1 × 10^6^ cells/well). When culturing for 12, 24, and 48 h, the medium was refreshed as serum-free medium and cells were added with 10 µL CCK-8 solution (CK04; Dojindo Molecular Technologies, Kumamoto, Japan) and cultured for 2 h at 37°C. The absorbance at 450 nm was obtained using a microplate reader (Multiskan MK3; Thermo Fisher Scientific).

### 2.9 Transwell assay

VSMCs were resuspended in serum‐free DMEM, and the cell density of the suspension was adjusted to 2.5 × 10^5^ cells/mL. The Transwell chambers (pore size of 8 μM; Coring Inc., NY, USA) were placed on a 24-well plate, 0.2 mL of cell suspension was added to the upper chamber, and 600 μL complete medium was added to the lower chamber. After the cell culture was continued for 24 h, non-migrated VSMCs were removed. Then, the remaining VSMCs attached on the lower surface of the Transwell membranes were fixed with 4% paraformaldehyde for 20 min and stained with 0.5% crystal violet solution. Subsequently, the stained VSMCs were counted under an inverted microscope (Olympus).

### 2.10 Flow cytometry

Apoptosis of VSMCs was detected by the AnnexinV–fluorescein isothiocyanate/propidium iodide (PI) double staining method. After 48 h of cell transfection, the cells were trypsinized, collected, and seeded in a six‐well plate at 2 × 10^6^ cells/well. Cell culture was continued for 24 h, and the medium was discarded. Cells were washed using pre-cooled PBS twice and then suspended in 1× binding buffer. Subsequently, cells were reacted with 5 μL of Annexin V and 1 μL of PI working solution for 15 min and added with 300 μL of binding buffer. The apoptosis was detected by flow cytometry within 1 h.

### 2.11 Intracellular reactive oxygen species (ROS) assay

Dichlorodihydrofluorescein diacetate (DCFH-DA) was adopted to evaluate ROS generation. Briefly, VSMCs were incubated with 10 mM DCFH-DA at 37°C for 15 min. After being washed three times with PBS, microscopic fluorescent images were observed under a fluorescent microscope (Olympus).

### 2.12 Luciferase reporter assay

The wild type or mutant type of TRAF6 3′UTR containing the binding site of miR-146-5p was subcloned into the pmirGLO vector (Promega, Madison, WI, USA) to generate TRAF6 3′UTR-Wt/Mut reporter. Next, pmirGLO vectors carrying TRAF6 3′UTR and miR-146-5p mimics were co-transfected into human VSMCs using Lipofectamine 2000 (Invitrogen). Luciferase Reporter Assay System (Promega) was applied to test luciferase activity levels.

### 2.13 Statistical analysis

GraphPad Prism software 6.0 was utilized to analyze data and all data are exhibited as mean ± standard deviation. *T* test was applied for comparison between two groups. The differences between multiple groups were compared by ANOVA and Tukey’s *post hoc* analysis. Only *p* < 0.05 was statistically significant.

## 3 Results

### 3.1 Ox-LDL treatment induces VSMC calcification and upregulates TRAF6 level in VSMCs

**Figure 1:**
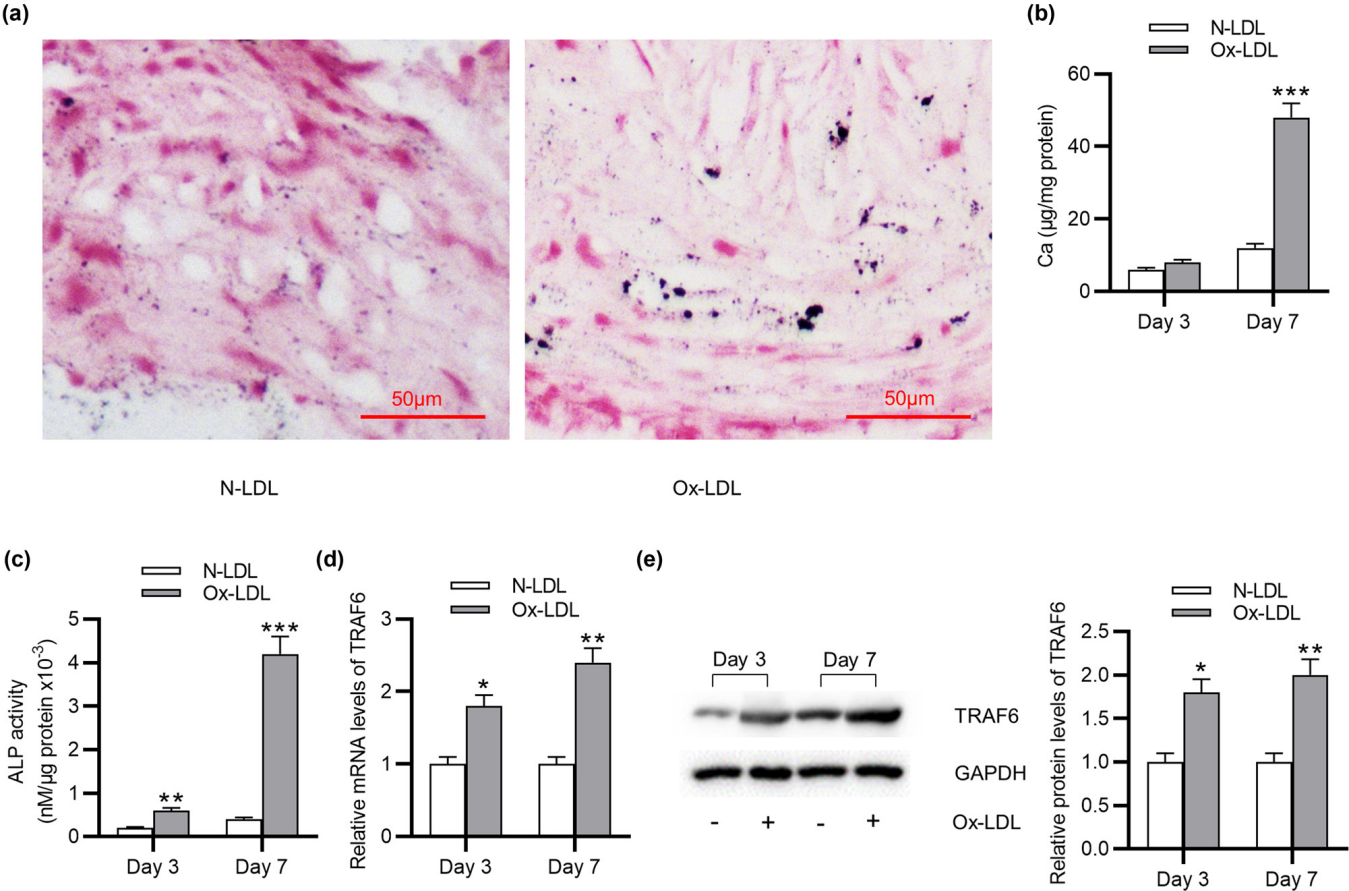
Ox-LDL treatment induces VSMC calcification and upregulates TRAF6 expression in VSMCs. (a) Mineral deposition in VSMCs treated with N-LDL or Ox-LDL was examined by von Kossa staining. (b) Calcium content in VSMCs after N-LDL or Ox-LDL treatment was measured at 3 and 7 days. (c) Osteogenic differentiation of VSMCs treated with N-LDL or Ox-LDL was assessed at 3 and 7 days by ALP activity analysis. (d) TRAF6 mRNA level in VSMCs treated with N-LDL or Ox-LDL was detected at 3 and 7 days using RT-qPCR. (e) TRAF6 protein level in VSMCs treated with N-LDL or Ox-LDL was detected at 3 and 7 days using western blotting. ^*^ *p* < 0.05, ^**^ *p* < 0.01, ^***^ *p* < 0.001.

To explore the effects of Ox-LDL on VSMC calcification, VSMCs were treated with 50 μg/mL Ox-LDL or N-LDL for 7 days. According to von Kossa staining, a significant elevation in mineral deposition was detected in Ox-LDL-stimulated VSMCs compared with N-LDL-treated VSMCs (Figure 1a). Next, calcium content was measured. At 3 days, neither N-LDL nor Ox-LDL significantly affected calcium content in VSMCs (Figure 1b). At 7 days, calcium content in VSMCs was markedly increased by Ox-LDL compared with N-LDL (Figure 1b). Osteogenic differentiation of VSMCs was assessed by ALP activity analysis. As Figure 1c demonstrates, at 3 days, ALP activity in VSMCs was slightly increased by Ox-LDL treatment. At 7 days, Ox-LDL-treated VSMCs exhibited significantly elevated ALP activity compared with N-LDL-treated VSMCs (Figure 1c), suggesting that Ox-LDL treatment induced osteogenic differentiation of VSMCs. We further tested the effects of Ox-LDL on TLR4 expression. TLR4 mRNA expression was significantly increased at 3 and 7 days in Ox-LDL-treated cells compared with N-LDL-treated cells (Figure 1d). Consistently, western blotting analysis also showed that Ox-LDL upregulated TLR4 protein expression in VSMCs (Figure 1e). Taken together, Ox-LDL treatment induces VSMC calcification and upregulates TRAF6 expression in VSMCs.

### 3.2 TRAF6 knockdown inhibits VSMC calcification induced by Ox-LDL

**Figure 2:**
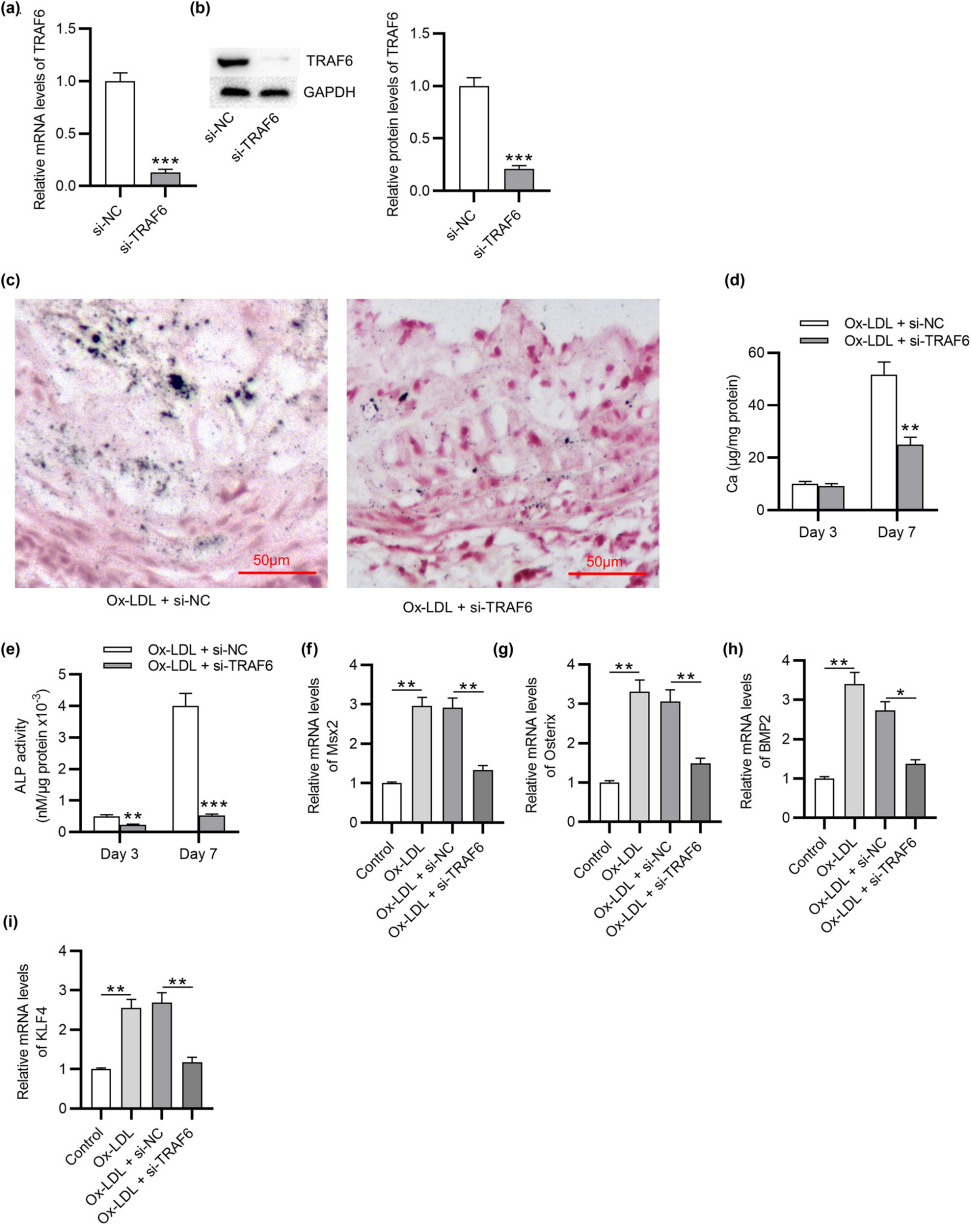
TRAF6 knockdown inhibits VSMC calcification induced by Ox-LDL. (a) TRAF6 mRNA level in Ox-LDL-treated VSMCs after transfection of si-NC or si-TRAF6 was tested by RT-qPCR. (b) TRAF6 protein level in Ox-LDL-treated VSMCs after transfection of si-NC or si-TRAF6 was tested by western blotting. (c) Mineral deposition in Ox-LDL-treated VSMCs after transfection of si-NC or si-TRAF6 was examined by von Kossa staining. (d) Calcium content in Ox-LDL-treated VSMCs after transfection of si-NC or si-TRAF6 was measured at 3 and 7 days. (e) Osteogenic differentiation of Ox-LDL-treated VSMCs after transfection of si-NC or si-TRAF6 was assessed at 3 and 7 days by ALP activity analysis. VSMCs were transfected with si-NC or si-TRAF6 for 48 h and then treated with 50 μg/mL Ox-LDL for 24 h. (f–i) The mRNA expression of osteogenic differentiation-related genes including Msx2, Osterix, BMP2, and KLF4 in Ox-LDL-treated VSMCs after transfection of si-NC or si-TRAF6 was measured by RT-qPCR. ^*^ *p* < 0.05, ^**^ *p* < 0.01, ^***^ *p* < 0.001.

To investigate the role of TRAF6 in VSMC calcification, Ox-LDL-treated VSMCs were transfected with si-NC or si-TRAF6, showing that mRNA and protein expression of TRAF6 was downregulated in VSMCs after transfection of si-TRAF6 (Figure 2a and b). von Kossa staining revealed that mineral deposition in VSMCs elevated by Ox-LDL was reduced after TRAF6 silencing (Figure 2c). TRAF6 knockdown also attenuated the increase in calcium content in VSMCs under Ox-LDL condition (Figure 2d). Moreover, Ox-LDL-induced elevation in ALP activity in VSMCs was counteracted by TRAF6 inhibition (Figure 2e). RT-qPCR showed that Ox-LDL increased the mRNA expression of osteogenic differentiation-related genes including Msx2, Osterix, BMP2, and KLF4 in VSMCs, while TRAF6 knockdown reversed these effects (Figure 2f and i). Overall, TRAF6 downregulation suppresses calcification and osteogenic differentiation in Ox-LDL-stimulated VSMCs.

### 3.3 TRAF6 knockdown inhibits VSMC proliferation, migration, inflammation, and ROS and accelerates apoptosis

**Figure 3:**
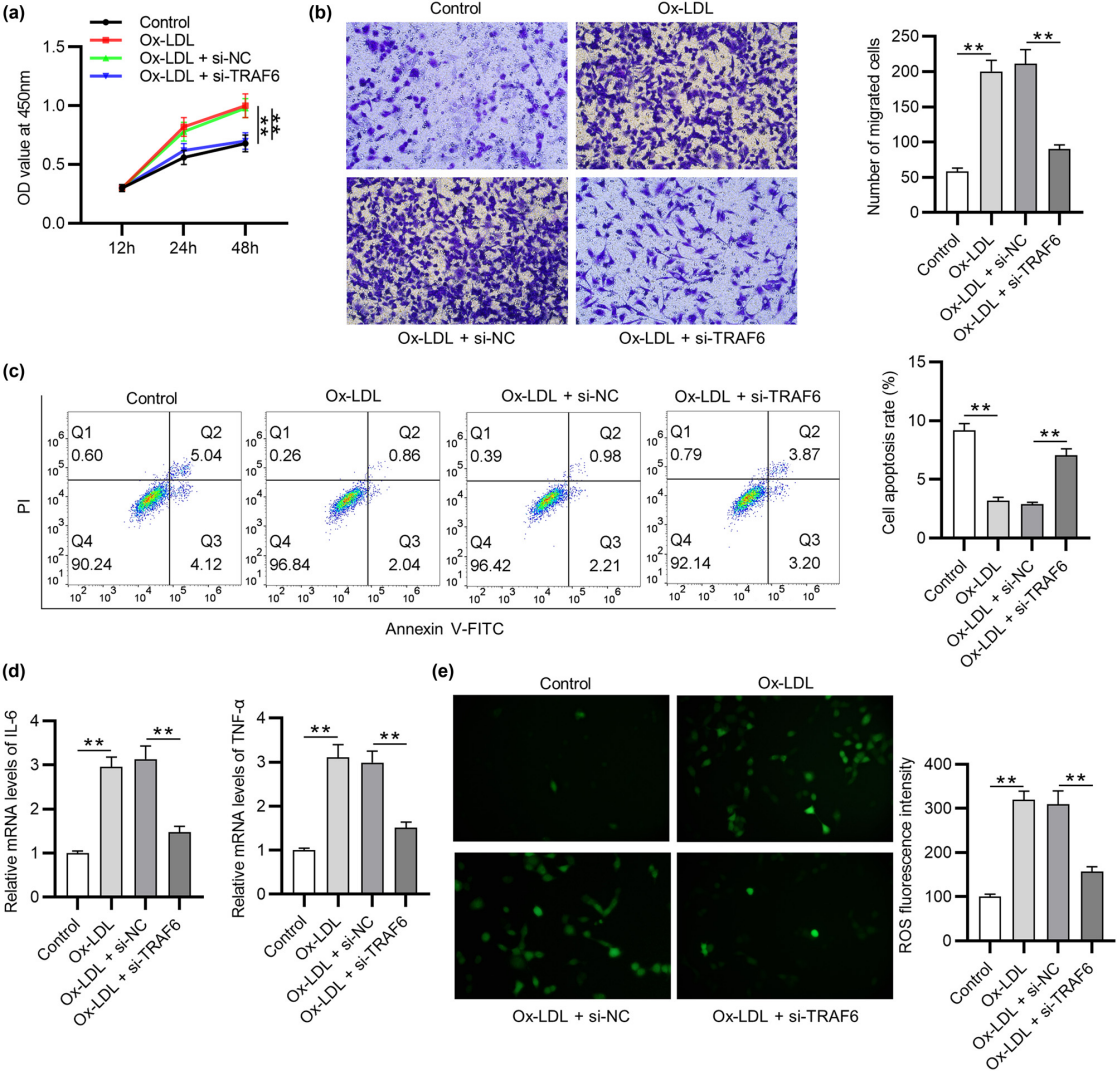
TRAF6 knockdown inhibits VSMC proliferation, migration, inflammation, and ROS and accelerates apoptosis. (a) The viability of Ox-LDL-treated VSMCs was detected by CCK‐8 assay. (b) The migration of Ox-LDL-treated VSMCs was assessed by Transwell assay. (c) Flow cytometry was applied to detect the apoptosis of Ox-LDL-treated VSMCs. (d) RT-qPCR was used to measure the mRNA expression of IL-6 and TNF-α in Ox-LDL-treated VSMCs. (e) DCFH-DA-labelled (green) ROS and density of ROS production in Ox-LDL-treated VSMCs were detected. ^**^ *p* < 0.01.

Next, the function of TRAF6 in ox‐LDL‐loaded VSMCs was further investigated. The results of CCK8 assay demonstrated that after downregulating the expression of TRAF6, the proliferation in Ox-LDL-treated VSMCs was decreased (Figure 3a). Transwell assay was applied to evaluate the migration of VSMCs induced by Ox-LDL (Figure 3b). The migration ability was enhanced in Ox-LDL-stimulated VSMCs compared with the control group and was decreased after transfection of si-TRAF6, confirming that TRAF6 knockdown inhibited the migration of VSMCs. Flow cytometry showed that Ox-LDL reduced the apoptosis of VSMCs, while TRAF6 knockdown restored the apoptosis in Ox-LDL-stimulated VSMCs (Figure 3c). Inflammation response after Ox-LDL treatment was assessed (Figure 3d). The levels of IL-6 and TNF-α were significantly elevated in OX-LDL-stimulated VSMCs. The inflammatory factor levels were reduced after transfection of si-TRAF6, suggesting that TRAF6 knockdown had inhibitory effects on inflammatory factor generation. ROS was detected to evaluate the oxidative stress (Figure 3e). As shown, ROS generation was higher in the OX-LDL-induced group or the OX-LDL-induced group transfected with si-NC than in the control group. The enhanced generation of ROS was attenuated in the OX-LDL-induced group transfected with si-TRAF6. Overall, TRAF6 downregulation suppresses proliferation, migration, inflammation, and ROS and accelerates apoptosis in OX-LDL-stimulated VSMCs.

### 3.4 TRAF6 is targeted by miR-146-5p

**Figure 4:**
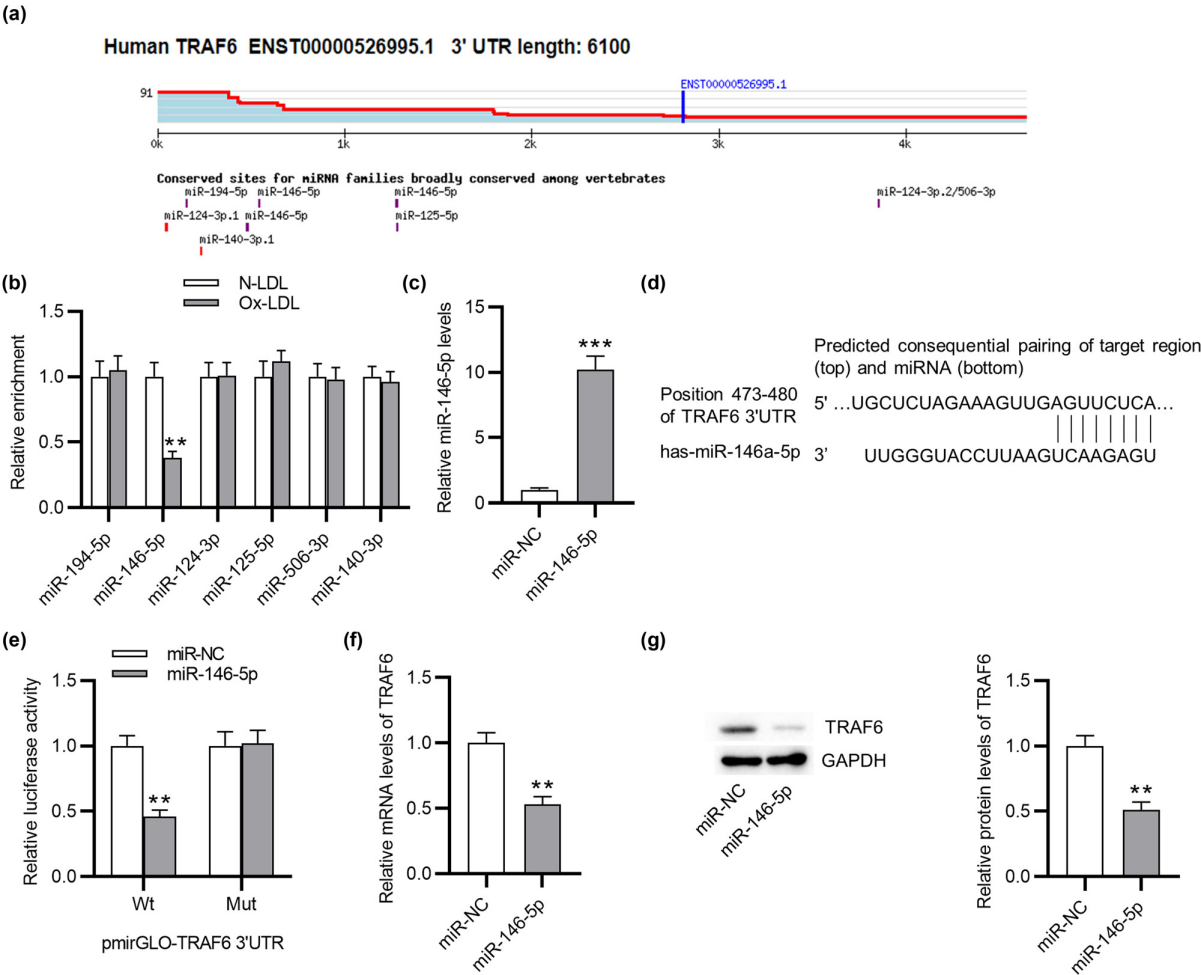
TRAF6 is targeted by miR-146-5p. (a) Potential upstream miRNAs (miR-194-5p, miR-146-5p, miR-124-3p, miR-125-5p, miR-506-3p, and miR-140-3p) of TRAF6 were predicted by Targetsan. (b) The expression of potential upstream miRNAs in VSMCs treated with N-LDL or Ox-LDL was detected by RT-qPCR. (c) Transfection efficiency of miR-146-5p mimics was evaluated by RT-qPCR. (d) A binding site of miR-146-5p in TRAF6 was predicted by Targetsan. (e) Interaction between miR-146-5p and TRAF6 was confirmed by luciferase reporter assay. (f–g) RT-qPCR and western blotting were performed to examine the mRNA and protein levels of TRAF6 in VSMCs after miR-146-5p overexpression. ^**^ *p* < 0.01, ^***^ *p* < 0.001.

The potential upstream miRNAs (miR-194-5p, miR-146-5p, miR-124-3p, miR-125-5p, miR-506-3p, and miR-140-3p) of TRAF6 were predicted by Targetsan (Figure 4a). Next, to identify which miRNA can bind to TRAF6, subsequent experiments were conducted. As Figure 4b indicates, Ox-LDL treatment only decreased miR-146-5p level in VSMCs. miR-146-5p was upregulated in VSMCs after transection of miR-146-5p mimics (Figure 4c). A binding site of miR-146-5p in TRAF6 was predicted by Targetsan (Figure 4d). Luciferase reporter assay exhibited that miR-146-5p overexpression decreased the luciferase activity of TRAF6 3′UTR-Wt compared with that of TRAF6 3′UTR-Mut, further confirming interaction between miR-146-5p and TRAF6 3′UTR (Figure 4e). In addition, the mRNA and protein levels of TRAF6 were reduced by miR-146-5p overexpression in VSMCs (Figure 4f and g). All in all, TRAF6 a target of miR-146-5p.

### 3.5 TRAF6 elevation counteracts the inhibitory effects of miR-146-5p upregulation on VSMC calcification induced by Ox-LDL

**Figure 5:**
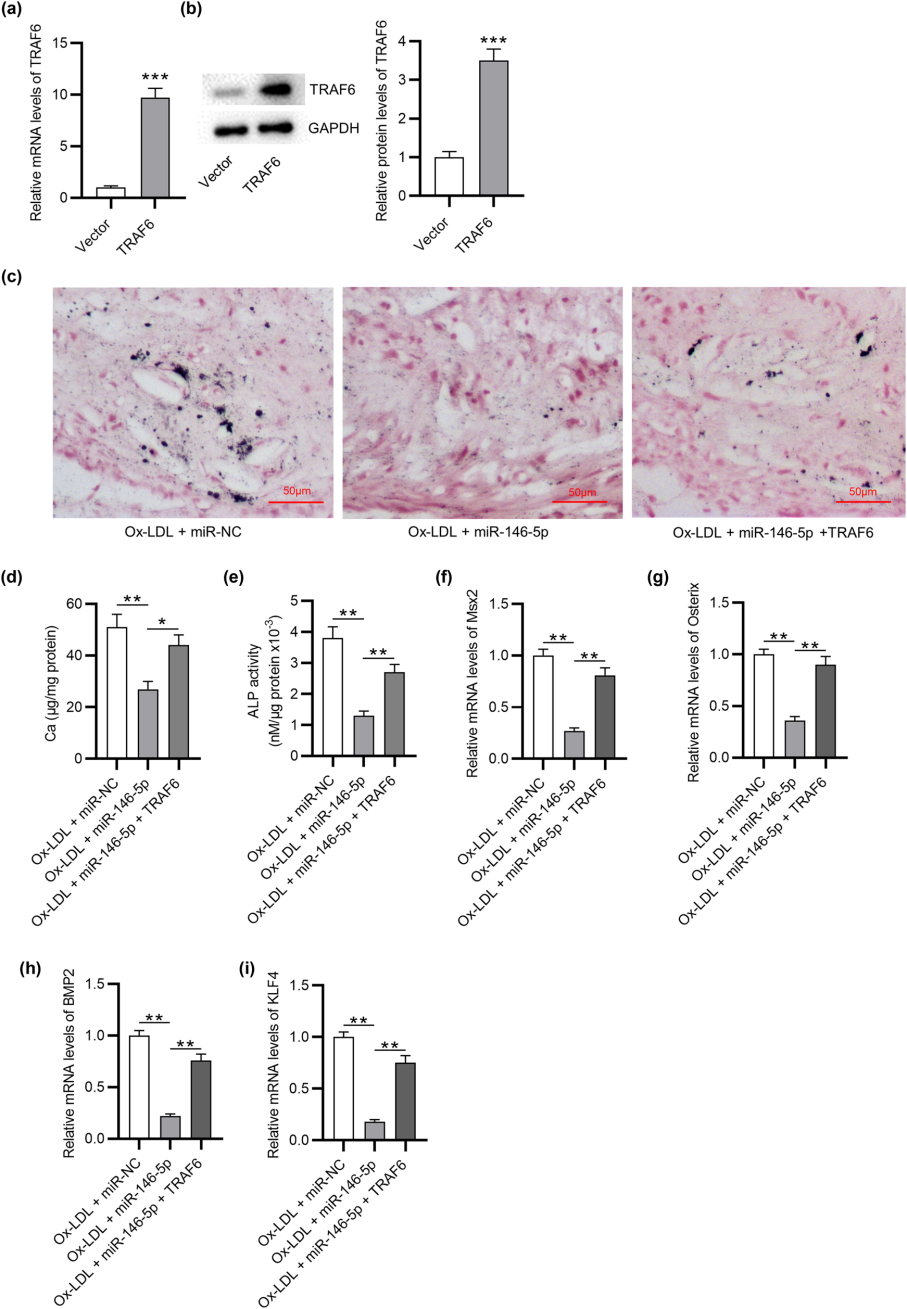
TRAF6 elevation counteracts the effects of miR-146-5p upregulation on VSMC calcification induced by VSMCs. (a and b) TRAF6 mRNA and protein levels in Ox-LDL-stimulated VSMCs after overexpressing TRAF6 were tested by RT-qPCR and western blotting. VSMCs were divided into Ox-LDL + miR-NC group, Ox-LDL + miR-146-5p group, and Ox-LDL + miR-146-5p + TRAF6 group. (c) Mineral deposition in each group was examined by von Kossa staining. (d) Calcium content in each group was measure. (e) ALP activity analysis was performed to assess the osteogenic differentiation of VSMCs each group. (f–i) The mRNA expression of osteogenic differentiation-related genes including Msx2, Osterix, BMP2, and KLF4 in each group was tested by RT-qPCR. ^*^ *p* < 0.05, ^**^ *p* < 0.01, ^***^ *p* < 0.001.

To validate the role of the miR-146-5p/TRAF6 regulatory axis in Ox-LDL-induced calcification, a series of rescue experiments were carried out. After transfection of pcDNA3.1 vectors overexpressing TRAF6, the mRNA and protein levels of TRAF6 in VSMCs under Ox-LDL condition were significantly upregulated (Figure 5a and b). von Kossa staining suggested that upregulated miR-146-5p-induced inhibition in mineral deposition was attenuated by TRAF6 elevation in Ox-LDL-treated VSMCs (Figure 5c). miR-146-5p overexpression decreased calcium content in VSMCs under Ox-LDL condition, which was reversed by elevated TRAF6 (Figure 5d). The suppression in ALP activity caused by miR-146-5p overexpression was mitigated by TRAF6 upregulation in VSMCs with Ox-LDL stimulation (Figure 5e). Moreover, TRAF6 upregulation reversed the inhibitory effects of miR-146-5p overexpression on the mRNA expression of Msx2, Osterix, BMP2, and KLF4 in Ox-LDL-treated VSMCs (Figure 5f and i). These data suggested that the inhibitory effects of miR-146-5p upregulation on VSMC calcification induced by Ox-LDL are reversed by TRAF6 overexpression.

### 3.6 TRAF6 overexpression reverses the effects of miR-146-5p on VSMC overexpression proliferation, migration, apoptosis, inflammation, and ROS

**Figure 6:**
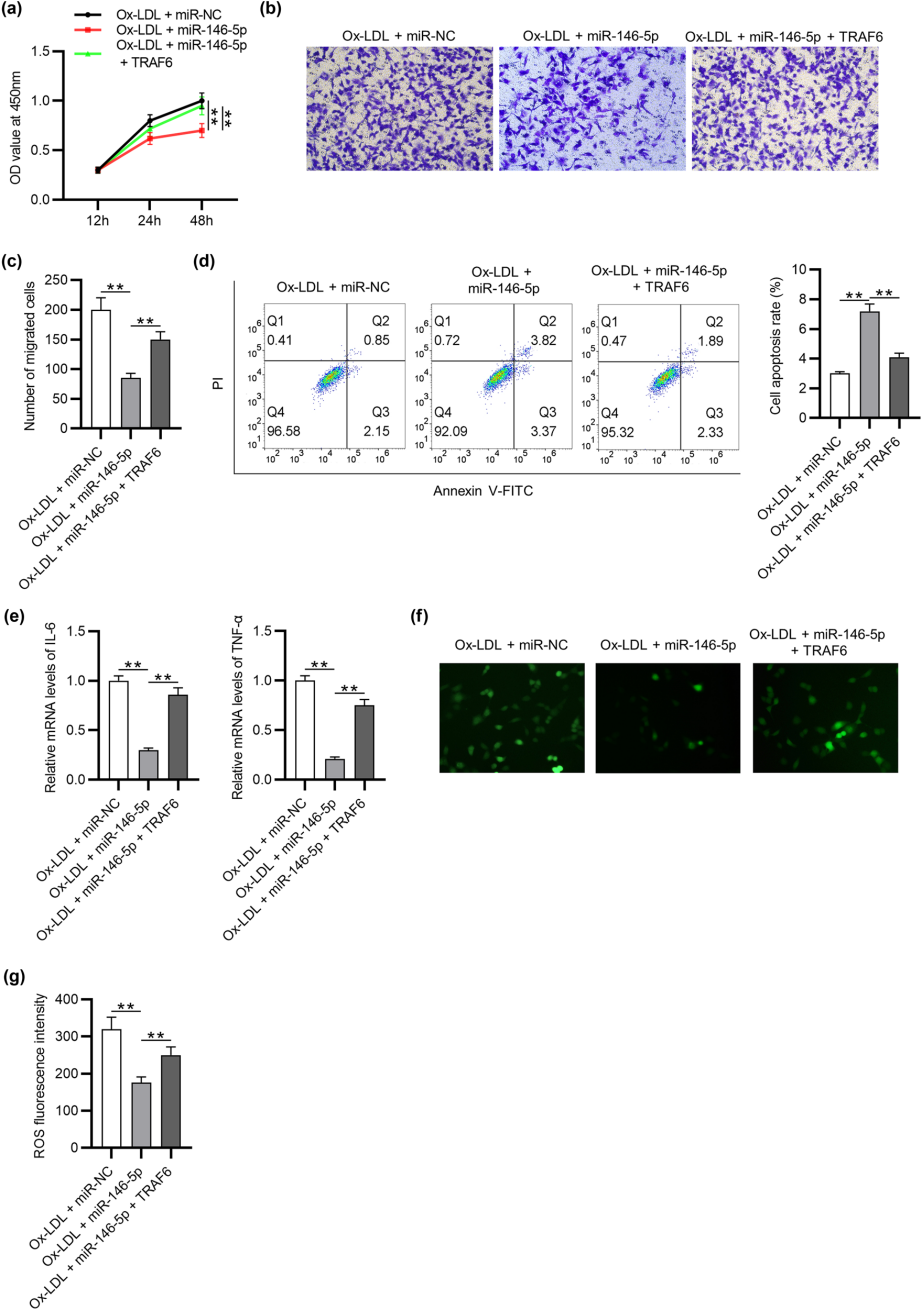
TRAF6 overexpression reverses the effects of miR-146-5p on VSMC overexpression proliferation, migration, apoptosis, inflammation, and ROS. (a–c) The viability and migration of VSMCs in each group were detected by CCK‐8 and Transwell assays. (d) Flow cytometry was applied to detect the apoptosis of VSMCs in each group. (e) The mRNA expression of IL-6 and TNF-α in each group was measured by RT-qPCR. (f and g) DCFH-DA-labelled (green) ROS and density of ROS production in each group were detected. ^**^ *p* < 0.01.

The results of CCK8 and Transwell assays showed that, in Ox-LDL-treated VSMCs, TRAF6 overexpression notably reversed the miR‑146-5p–mediated reduction in the proliferation and migration of VSMCs (Figure 6a and c). Overexpression of miR‑146-5p significantly inhibited the VSMC apoptosis induced by Ox-LDL; however, this effect was attenuated by TRAF6 overexpression (Figure 6d). Furthermore, the suppressed inflammation and ROS generation mediated by miR‑146-5p were significantly restored by TRAF6 overexpression (Figure 6e and g). These results showed that miR‑146-5p regulates VSMC overexpression proliferation, migration, apoptosis, inflammation, and ROS by targeting TRAF6.

## 4 Discussion

Atherosclerosis is a chronic inflammatory disorder related to cardiovascular diseases [26]. Vascular calcification is a prominent manifestation of atherosclerosis [27]. As an independent risk factor of atherosclerosis, Ox-LDL can carry a large amount of cholesterol into the vascular intima and easily cause thrombosis [28]. Many reports have demonstrated that Ox-LDL is implicated in vascular calcification. For example, Ox-LDL contributes to VSMC calcification, which is mediated by nuclear factor of activated T cells [29]. Ox-LDL facilitates VSMC calcification via regulating ceramide signaling [23]. Here, to identify the effects of Ox-LDL on vascular calcification of VSMCs, Ox-LDL was applied to stimulate VSMCs. We found that Ox-LDL induced VSMC calcification after 7 days. Next, the mRNA and protein expression of TRAF6 was found to be overexpressed in Ox-LDL-treated VSMCs. After downregulating TRAF6, Ox-LDL-caused elevation in mineral deposition, calcium content, and ALP activity in VSMCs was attenuated. In addition, TRAF6 was reported to promote cell proliferation and migration in many cancers [30,31]. TRAF6 is an essential mediator of CD40-activated pro-inflammatory pathways, such as NF-kB [32]. TRAF6 could aggravate inflammatory response and oxidative stress under many pathological conditions [33,34]. Interestingly, in this study, we found that TRAF6 knockdown inhibited VSMC proliferation, migration, inflammation, and ROS and accelerated apoptosis. These findings showed that TRAF6 may promote the progression of atherosclerosis.

As small noncoding RNAs with 19–25 nucleotides in length [35], miRNAs directly bind to the 3′UTR of target gene sequences to degrade mRNA or inhibit protein translation [36]. Accumulating studies have demonstrated that miRNAs are involved in atherosclerosis. For example, miR-141-5p reduces the inflammation, proliferative, and migrative abilities of VSMCs by inhibiting the HMGB1/NF-κB pathway [37]. miR-146b-3p targets PIK3CG to repress the phenotype switch, proliferation, and migration of VSMCs [38]. miR-33a-5p inhibits ox-LDL-induced VSMC calcification via binding to METTL3 during atherosclerosis development [39]. In the current study, miR-146-5p was identified as the upstream miRNA of TRAF6. miR-146-5p is a cluster of miR-146 family. miR-146 members were reported to participate in the development of atherosclerosis. miR-146a-5p is dysregulated in animal models of atherosclerosis [40]. LPS induces CXCL16 expression through the TLR4 pathway mediated by miR-146a- in human umbilical vein endothelial cells [41]. In mice with no reduction in plasma lipid, miR-146a enrichment mitigates atherosclerosis and the activation of monocyte/macrophage [42]. Here, miR-146-5p negatively regulated mRNA and protein levels of TRAF6 in VSMCs. In rescue assays, miR-146-5p upregulation suppressed mineral deposition, calcium content, and ALP activity in Ox-LDL-stimulated VSMCs, as well as inhibited VSMC proliferation, migration, inflammation, and ROS and accelerated apoptosis, and these changes were reversed by TRAF6 overexpression.

In conclusion, miR-146-5p restrains calcification of VSMCs by suppressing TRAF6, which may offer a therapeutical target for atherosclerosis treatment. However, this study should be improved to get more accurate results. Since some pathways (e.g., NF-κB signaling [43], Notch signaling pathway [44], and PI3K/Akt signaling pathway [45]) were demonstrated to participate in atherosclerosis development; whether the miR-146-5p/TRAF6 axis can regulate these pathways in Ox-LDL-treated VSMCs should be investigated. Moreover, *in vivo* studies should be conducted in the future.
